# Comparison of plasma PARK7 and NDKA diagnostic value in acute stroke

**DOI:** 10.2144/fsoa-2018-0080

**Published:** 2019-02-12

**Authors:** Dieudonne Steve M Tulantched, Zhao Min, Wang-Xiao Feng

**Affiliations:** 1 Department of Emergency Medicine, Shengjing Hospital of China Medical University, Shenyang 110004, PR China

**Keywords:** acute stroke, biomarkers, correlation, diagnostic value, hemorrhagic stroke, ischemic stroke, NDKA, PARK7, plasma, subarachnoid hemorrhage

## Abstract

**Aim:**

In this prospective case–control study we aimed to compare diagnostic value of plasma PARK7 and NDKA in early diagnosis of acute stroke and evaluate the validated diagnostic values of PARK7 and NDKA in an independent patient cohort. We then assessed the quantitative relationship between the release of these markers: stroke severity and time. Blood samples were drawn upon hospital admission and 14 days later. PARK7 and NDKA concentrations were measured using an ELISA.

**Results:**

The expression of PARK7 (area under the curve [AUC] = 0.897) in acute stroke patients was more significant than in controls, relative to the NDKA expression (AUC = 0.462); p < 0.05. Their expressions were not related to the clinical characteristics of both groups; p > 0.05.

**Conclusion:**

Even though both markers cannot differentiate stroke etiologies (ischemic or hemorrhagic), plasma PARK7 has better diagnostic value than NDKA for early diagnosis of stroke. 72 plasma samples obtained from acute stroke patients and 78 plasma samples collected from non-stroke patients were analyzed in this study.

## Lay abstract

The gold standard diagnostic tool for stroke remains to be neuroimaging (CT or MRI), but it can be performed only in sophisticated hospitals. Biomarkers, including PARK7 and NDKA, have been suggested for early diagnosis of patients with stroke. The aim of this study was to determine which one has more diagnostic value for acute stroke. Two groups were included: one contained patients with stroke and the other patients without. After comparing the diagnostic values of both biomarkers in both groups, PARK7 seemed more reliable than NDKA.

According to recent surveys, the incidence rate of stroke is up to 119 patients per 100,000 person-years in high-risk regions like New Zealand, and reaches 345.1/100,000 person-years in PR China. In all epidemiologic stroke studies, the age-standardized mortality is significant, around 114.8/100,000 person-years in PR China, making stroke the second leading cause of mortality worldwide [1,2].

Although there has been considerable advancement in the understanding of its pathophysiology, efficient therapies are still needed to reduce the mortality and disability rate due to stroke. Stroke biomarkers may potentially aid in diagnosis, identification of intracerebral injury, evaluation of severity and prognosis of treatment [3–5].

Biomarkers are biological molecules identified and measured by biochemical techniques using blood, urine, cells or tissues that assist in the diagnosis, prognosis and physiology of a disease or the risk factor for this disease [6]. Timely diagnosis of stroke is important in clinical practice for efficient treatment. The measurements of circulating biomarkers may facilitate early diagnosis of stroke. Many molecules were studied to suggest such prediction for stroke [7–11], but most of them have shown some weakness, as their release occurs at a late stage after stroke onset [11].

The newly identified biomarkers NDKA and PARK7 are brain-specific and easily detectable upon exceeding normal values at the onset of stroke, suggesting that they could be reliable biomarkers for early diagnosis of stroke [11,12]. A recent study has shown the role of PARK7 and NDKA in stroke pathophysiology: PARK7 acting as an antioxidant, chaperoning molecules and transcriptional regulator in stroke, protecting from neuronal cell deaths. As an enzyme, NDKA has also been shown to catalyze stroke-associated reactions [12]. The objective of this study was to compare the diagnostic values of circulating PARK7 and NDKA in plasma of stroke patients, and evaluate their use and limitations as biomarkers for early stroke diagnosis.

## Patients & methods

### Clinical specimens

72 stroke patients (admitted to the emergency department of Shengjing Hospital affiliated to China Medical University, Shenyang, PR China) and 78 patients without stroke (patients with other illness, as controls) were enrolled in this study. The protocol was approved by the Medical Research and New Technology Ethics Committee of Shengjing Hospital affiliated to China Medical University.

### Selection criteria

Stroke patients were recruited in accordance with the guidelines: patients with obvious manifestations of stroke, positive stroke neuroimaging and clinical confirmation by a neurologist were diagnosed as stroke cases. Patients with transient ischemic attack were excluded due the lack of proper characterization of the disease at admission. In order to avoid bias, we excluded patients with mimic stroke and neurologic disorders.

### Plasma collection & storage

4 ml of venous blood samples were collected from all individuals using sodium citrate-coated tubes. The median time of first collection from stroke onset was 17 h (interquantile range [IQR] = 22; mean of 17.92 ± 17.717). The second time point collection was on day 14. Depending on the progression of the disease and management of patients, we collected blood from 20 patients on day 14 after admission. Plasma was obtained by centrifugation of whole blood at 1500xg for 15 min at 4°C in EP tubes and stored at -80 until laboratory analysis.

### PARK7 & NDKA ELISA

The plasma levels of NDKA and PARK7 were measured by ELISA at the Shengjing Hospital's medical and pharmaceutical research laboratory in Benxi. Abcam (London, UK) ab215535 Human PARK7 SimpleStep ELISA kit and Mlbio (Good ELISA kit producers, Shanghai, PR China) Human NDKA kit were procured and used for the assay of PARK7 and NDKA, respectively. The experiments were conducted following manufacturers’ instructions.

Succinctly, 50 μl of diluted plasma was used for assays and the dilution ratios were twofold and fivefold for PARK7 and NDKA, respectively. Biomarker concentrations were calculated from their respective absorbance values determined at 450 nm. The results were expressed as ng/ml.

### Statistical analysis

Statistical Package for Social Sciences (IBM SPSS version 20) was used for statistical analysis and graph drawing. Data are presented as mean ± standard deviation (x ± SD), unless otherwise noted. The significance of difference in clinical characteristics between stroke patients and controls was tested using Chi Square test. Both PARK7 and NDKA levels were measured at two time points (day 1 at admission and day 14) and compared using Wilcoxon's Signed Rank Test. The Mann-Whitney U test was used to compare the expression of both biomarkers between stroke patients and controls. Kruskal-Wallis test and Dunn's multiple comparison were used to discriminate the expression of these biomarkers in ischemic stroke (IS), hemorrhagic stroke (HS) and subarachnoid hemorrhage (SAH). Receiver-operating characteristic (ROC) curves were established to discriminate between stroke patients and controls. The association of biomarker levels with National Institute for Health Stroke Scale (NIHSS) was elucidated using Spearman correlation. Our attempt at comparing the diagnostic values of plasma PARK7 and NDKA was completed with ROC comparison using MedCalc 17.9.7 (Hanley & McNeil). All p-values were two-tailed and p < 0.05 was statistically significant.

## Results

### Clinical characteristics

Our study enrolled 72 stroke patients and 78 controls (nonstroke patients). The clinical characteristics of our sample individuals are listed in Table 1. There was no significant difference in the characteristics between stroke and control groups (p > 0.05). The mean age of stroke patients was 59.4 ± 14.1 years and the mean age of controls 53.7 ± 19.7 years old (p = 0.109). Both groups were predominantly male (46/26 in stroke group and 45/33 in control group; p = 0.121).

**Table 1.** **Clinical characteristics of the study population.**

**Population characteristics**			**Stroke patients, n = 72**	**Control, n = 78**	**p-value**
Age	Years	Mean ± SD	59.4 ± 14.1	53.7 ± 19.7	0.109

		Min–max	25–89	20–91	

Sex	Female	n (%)	26 (36%)	33 (42%)	0.121

	Male	n (%)	46 (64%)	45 (58%)	

Diabetes		n (%)	25 (34.7%)	34 (43.6%)	0.267

Hyperlipidemia		n (%)	10 (13.9%)	12 (15.4%)	0.796

Smokers		n (%)	35 (48.6%)	33 (42.3%)	0.438

Hypertension		n (%)	37 (51.4%)	29 (43.9%)	0.08

Systolic blood pressure	mmHg	Mean ± SD	150.40 ± 29.72	140.33 ± 30.97	0.106

Distolic blood pressure	mmHg	Mean ± SD	88.50 ± 15.67	85.23 ± 16.16	0.328

Types of stroke	Hemorrhagic	n (%)	26 (36.1%)		

	Ischemic	n (%)	39 (54.1%)		

	Subarachnoid hemorrhage	n (%)	7 (9.8%)		

Without mention of biomarker levels, there were no significant differences in all clinical characteristics between the stroke (72) and control (78) groups (p > 0.05).

SD: Standard deviation.

### Diagnostic values of NDKA & PARK7 in stroke

**Figure 1.**
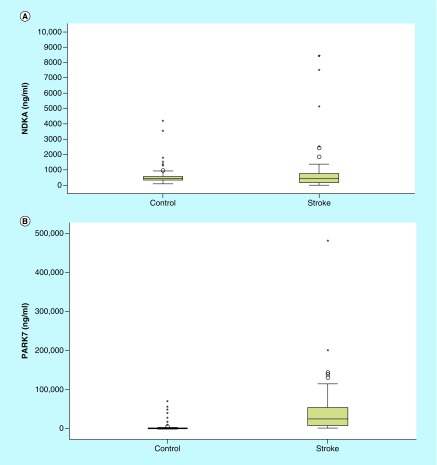
**Plasma levels of NDKA & PARK7 in stroke versus controls.** **(A)** Change in NDKA and PARK7 levels from day 1 to day 14 in stroke patients versus controls. Histogram of plasma NDKA level in stroke group and control group. Level of NDKA in stroke patients at admission time in emergency department. The plasma levels of NDKA were not significantly increased in stroke patients (n = 72) compared with controls (78); p > 0.05. **(B)** Histogram of plasma PARK7 level in stroke group and control group. Level of PARK7 in stroke patients at admission time at emergency department. The plasma levels of PARK7 were significantly increased in stroke patients (n = 72) compared with controls (78); *p = 0.000.

**Figure 2.**
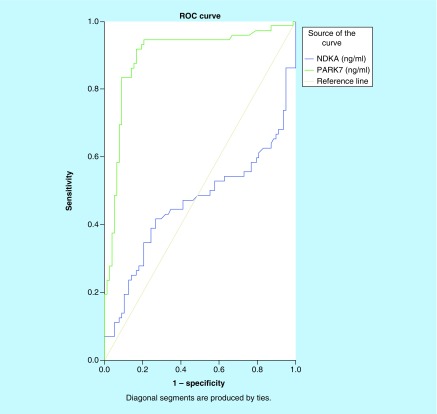
**Receiver-operating characteristic curves of plasma NDKA and PARK7 control versus stroke.** Comparison of the sensitivity and specificity for the diagnosis by plasma PARK7 and NDKA in stroke patients. Receiver-operating characteristic curves were drawn to evaluate the diagnostic values of PARK7 in comparison with NDKA. Following the area under the receiver-operating characteristic curve, PARK7 has greater diagnostic value than NDKA.

We investigated the differences in plasma PARK7 and NDKA levels between stroke patients and controls. As shown in Figure 1A & B, there was an increase in levels of both molecules in patients with stroke compared with the control patients. PARK7 was markedly elevated in stroke patients relative to controls (p < 0.001), while NDKA did not show such significance (p > 0.05). To evaluate the predictive power of circulating PARK7 and NDKA for stroke diagnosis, we performed ROC analysis for 72 patients with stroke. As displayed in Figure 2, we found that PARK7 had a higher diagnostic potential with an area under the curve (AUC) of 0.897 (95% CI: 0.841–0.954; p < 0.001) and an OR (odds ratio) of 1.087 (95% CI: 1.049–1.126; p < 0.001), while NDKA gave an AUC of 0.462 (95% CI: 0.365–0.560, p > 0.05) that corresponded to an OR of 0.882 (95% CI: 0.623–1.248).

**Figure 3.**
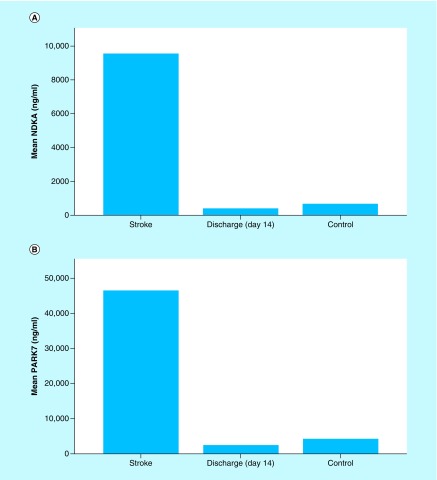
**Histograms of biomarker means in stroke patients at admission vs 14 days after admission vs controls.** **(A) Mean plasma NDKA levels in stroke patients at admission (day 1) versus at discharge (day 14) versus controls.**Presentation of mean levels of NDKA in stroke patients at day 1 and on day 14 in comparison with controls. Alteration of Plasma NDKA levels in stroke patients. On day 14 (n = 20), the measurement of NDKA returns to the baseline levels with values less than controls (n = 78), p = 0.007 (discharge on day 14 vs controls). **(B)** Mean plasma PARK7 levels in stroke patients at admission (day 1) versus at discharge (day 14) versus controls. Presentation of mean levels of PARK7 in stroke patients at day 1 and on day 14 in comparison with controls. Alteration of plasma PARK7 levels in stroke patients. PARK7 is markedly increased in plasma samples gathered at admission after stroke onset (within 24 h). On day 14 (n = 20), the measurement of PARK7 returns to the baseline levels with values similar to controls (n = 78); p > 0.05 (discharge on day 14 vs control).

On day 14, the plasma levels of both biomarkers were decreased to the baseline levels (p < 0.05). Especially for PARK7, the level at day 14 was not statistically different from controls level (p > 0.05) as shown in Figure 3A & B.

These results demonstrated that PARK7 may have more sensitivity and more specificity for stroke than NDKA.

### Correlations between PARK7 & NDKA plasma levels & stroke severity

The severity of neuronal dysfunctions in patients with stroke was evaluated according to NIHSS performed by a trained neurologist on admission and day 14 after stroke onset. The pairwise comparison of NIHSS in two time points demonstrated significant difference (p = 0.003), suggesting the gradual improvement in neurological functions among the stroke patients due to the implementation of efficient therapies or improvement of patients conditions. Although there was a decrease in plasma PARK7 and NDKA levels from admission time to day 14 (p < 0.05), the correlation analysis between these biomarkers and NIHSS at each time point showed no significant association (p > 0.05).

### Circulating PARK7 & NDKA to identify IS, HS & SAH

At admission, the level of each biomarker among patients with IS, HS and SAH was analyzed using nonparametric tests (Kruskal-Wallis H test followed by Dunn's multiple comparison p > 0.05/3). The results demonstrated that both molecules can similarly identify patients with the three types of stroke, there was no statistical difference in the capacity of PARK7 and NDKA to identify IS, HS or SAH (p > 0.0166).

**Figure 4.**
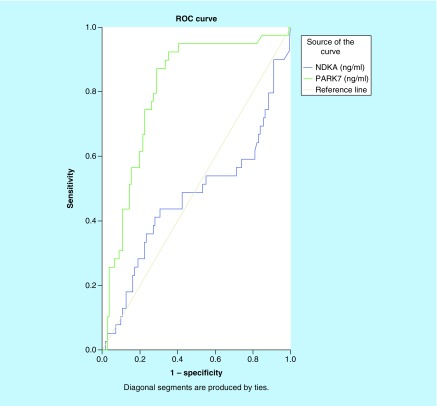
**Receiver-operating characteristic curves of plasma NDKA and PARK7 control versus ischemic stroke.** Comparison of the sensitivity and specificity for the diagnosis by plasma PARK7 and NDKA in ischemic stroke. Receiver-operating characteristic curves were drawn to evaluate the diagnostic values of PARK7 in comparison with NDKA. The PARK7 has greater area under the curve than NDKA.

**Figure 5.**
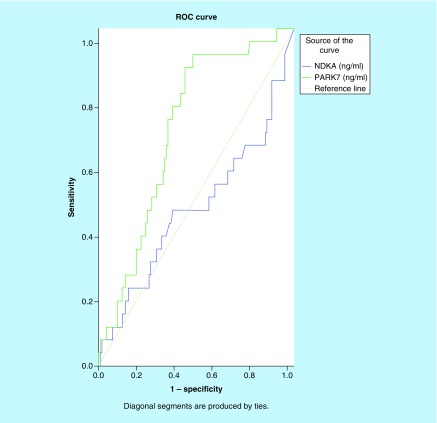
**Receiver-operating characteristic curves of plasma NDKA and PARK7 control versus hemorrhagic stroke.** Comparison of the sensitivity and specificity of the diagnosis by plasma PARK7 and NDKA in hemorrhagic stroke. Receiver-operating characteristic curves were drawn to evaluate the diagnostic values of PARK7 in comparison with NDKA. The PARK7 has greater area under the curve than NDKA.

As shown in Figure 4, the ROC curve was used to compare the diagnostic capacity of both biomarkers for IS. With an AUC of 0.806 (95% CI: 0.727–0.844; p < 0.001), PARK7 presented a good diagnostic accuracy while NDKA showed no diagnostic capacity for IS, AUC = 0.473 (95% CI: 0.355–0.590; p > 0.05). Other ROC curves were drawn to compare the diagnostic values of PARK7 and NDKA for HS. As shown in Figure 5, the results suggested PARK7 as better than NDKA with an AUC of 0.702 (95% CI: 0.605–0.800; p = 0.001). Plasma NDKA level showed no diagnostic accuracy for HS: AUC of 0.466 (95% CI: 0.330–0.603; p > 0.05).

**Figure 6.**
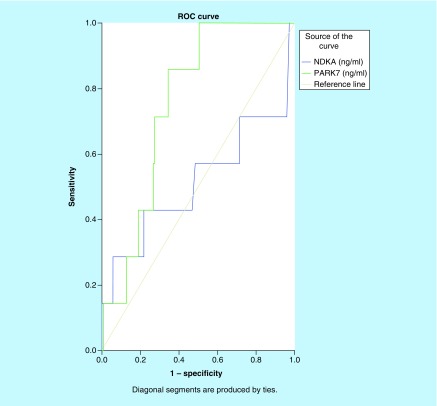
**Receiver-operating characteristic curves of plasma NDKA and PARK7 control versus subarachnoid hemorrhage.** Comparison of the sensitivity and specificity of the diagnosis by plasma PARK7 and NDKA in subarachnoid hemorrhage. Receiver-operating characteristic curves were drawn to evaluate the diagnostic values of PARK7 in comparison with NDKA. The PARK7 has greater area under the curve than NDKA.

Finally, for the comparison of diagnostic capacity of both markers for SAH, we drew another ROC curve (Figure 6). The results also suggested PARK7 as better than NDKA (AUC: 0.756 vs 0.515; p = 0.001 vs p > 0.05).

## Discussion

The aim of our study was to compare the diagnostic value of plasma PARK7 and NDKA. PARK7 and NDKA were suggested as potential biomarkers for stroke diagnosis in recent studies [12]. PARK7 plays multiple important roles in stroke; it acts as an antioxidant, a transcription regulator, a chaperone molecule and a degradation protein. PARK7, also called DJ-1, is sensitive to oxidation because of its cysteine residue (C106) that picks up the hydrogen peroxide. Its degree of oxidation determines the activity of PARK7. It is thus involved in protection of neurons. It also has a therapeutic potency in neurodegenerative disorders including IS in which it helps to reduce the size of an infarct [12–18]. However, PARK7 impairs bacterial clearance in patients with sepsis because of its counteractive role to the reactive oxygen species, a killer of inflammation and bacterial affection. It also acts in the IL-1/TNF-α pathway involved in many inflammations: pancreatitis, gastrointestinal disease and celiac disease [19–21]. In addition to being involved in the fertilization, PARK7 has also been demonstrated to play a role in the repair of nucleotides and nucleic acids [18,22,23].

The detection of NDKA in victims of brain injuries suggested plasma NDKA as a diagnostic marker of head trauma, IS, HS, SAH, transient ischemic attack and mild traumatic brain injury [24,25].

The findings of this study suggest that PARK7 is more reliable than NDKA both in sensitivity and specificity. PARK7 especially, having increased early on in stroke patients, remained high up to 3 days after stroke onset and decreased to normal baseline values on day 14. These findings are consistent with previous studies [11,26–29].

As shown in Table 2, the pairwise comparison of ROC curves for both plasma markers in each type of stroke (IS, HS, SAH) demonstrated that the diagnostic value of PARK7 is greater than that of NDKA.

**Table 2.** **Pairwise comparison of receiver-operating characteristic curves of PARK7 and NDKA.**

**Statistical variables**	**Statistical values**
**PARK7 ∼ NDKA for stroke**	

Difference between areas	0.360

Standard error	0.0564

95% CI	0.249–0.470

z statistic	6.381

Significance level	p < 0.0001

**PARK7 ∼ NDKA for ischemic stroke**	

Difference between areas	0.168

Standard error	0.0842

95% CI	0.00331–0.334

z statistic	1.999

Significance level	p = 0.0456

**PARK7 ∼ NDKA for hemorrhagic stroke**	

Difference between areas	0.279

Standard error	0.0699

95% CI	0.142–0.416

z statistic	3.983

Significance level	p = 0.0001

**PARK7 ∼ NDKA for subarachnoid hemorrhage**	

Difference between areas	0.241

Standard error	0.153

95% CI	– 0.0594 to 0.541

z statistic	1.572

Significance level	p = 0.1159

Pairwise comparison of receiver-operating characteristic curve of both plasma markers for stroke and each type of stroke (IS, HS, SAH), we see that PARK7 has more diagnostic value at the admission time than NDKA with p < 0.0001, p = 0.0456 and p = 0.0001 for all stroke diseases, IS and HS, respectively. Unfortunately, we did not find statistical difference between both molecules for SAH, p = 0.1159; more studies with larger population of SAH patients are needed.

CI: Confidence interval; HS: Hemorrhagic stroke; IS: Ischemic stroke; SAH: Subarachnoid hemorrhage.

PARK7 is then more reliable for early diagnosis of stroke; it can be useful for sorting out stroke patients in emergency medical services, helping for early correct triage of stroke patients. It may also help identify individuals at risk of early neurologic deterioration [30,31].

The identification of suitable stroke biomarkers is a great challenge nowadays; however, the results suggest some limitations for their application in stroke management [6]. Although PARK7 and NDKA play known roles in stroke pathophysiology [12], there are some problems facing their use in clinical routine:

- PARK7 and NDKA levels are similarly expressed in all stroke types, so they could not differentiate IS and HS. This result complements previous findings [11,26].
- At the disease level and at a given time point, both molecules lacked value for assessing the neuronal dysfunction severity (NIHSS) in acute stroke patients. This finding correlates to those from recent studies [26,29].
- NDKA levels may be different according to the laboratory technique used and the sampling time [11]. Since the median time in our study was 17 h after stroke onset, this might justify the slight different in reliability of NDKA compared with PARK7. With standard analytic kits, it remains less reliable for diagnosis of acute stroke [26,29].

We are aware of the limitations of our study. Our sample size might be small, but has some significance: it clarifies the diagnostic value of PARK7 over NDKA. The results obtained regarding PARK7 and NDKA reliability for acute stroke are complementary to latest findings [12,26,29] and our study suggests the superiority of PARK7 in diagnosis of acute stroke compared with NDKA. This is a new viewpoint, an important insight for more development in the biomarkers field. Nevertheless, the higher diagnostic value of PARK7 over NDKA will be improved upon in further large studies that are still ongoing in that field.

The median time that characterizes our population was about 17 h, the mean was 17.92 ± 17.717 h. Given that the treatment of stroke for better outcome (IS or HS) is time-dependent, such time is out of the traditional therapeutic window. However, this distribution is quite similar to the mean time of previous studies [11,26,33,34]. It might suggest that stroke patients do not reach hospital once the symptoms occur (wake-up strokes, for example); that critical stroke patients arrived at the emergency department late after being admitted to other hospitals or centers of lower levels; and that there are many factors putting stroke patients outside the window for time-dependent therapies, leading to a high rate of disability and mortality [32–34].

## Conclusion

Although the ultimate purpose of this study was to elucidate which biomarker of PARK7 and NDKA is more reliable for diagnosis of acute stroke, we recognize its limitation: small size of sample. In conclusion, PARK7 seems to be the most accurate biomarker for stroke diagnosis and might be involved in triage of patients with acute stroke. However, more studies including healthy volunteers or patients with other diagnoses initially suspected as stroke are needed to specify the usefulness of both biomarkers.

## Future perspective

We anticipate that PARK7 is usefulness in stroke diagnosis; its advantages over NDKA in prognosis, even in therapy of stroke patients, will be improved with further clinical research and animal experiments. In the future, we anticipate that more research in that direction will finally lead to the determination of a stroke biomarker as gold standard for diagnosis of stroke. Given that a significant proportion (25%) of patients who had not had stroke are referred to stroke services and belatedly diagnosed as having no stroke, the development of early diagnostic tools as well as blood biomarkers for stroke, which might help distinguish stroke from mimics, faces tremendous challenges; we anticipate that more research including stroke mimic subjects are already being run and will be conducted to compare blood marker levels between stroke patients and stroke mimic patients. Stroke mimics are diseases initially suspected as stroke at admission, diseases that imitate signs and symptoms of stroke (toxic-metabolic pathologies, seizure disorders, degenerative neurologic conditions and peripheral neuropathies): we anticipate that a biomarker that could accurately discriminate both groups (stroke vs stroke mimics or stroke vs patients initially suspected as stroke) would greatly impact stroke evaluation. The development of accurate analytic kits will also be a field of interest, focusing on the most reliable biomarkers [31,32,35–37].

**Summary points**

**Background**

- PARK7 and NDKA have been identified as playing role in stroke pathophysiology and as biomarkers for early diagnosis of stroke.
- Our aim was to compare the diagnostic value of PARK7 and NDKA in acute stroke.
- We aimed to answer to the question: is PARK7 more reliable than NDKA in acute stroke?

**Methods**

- 72 patients with stroke have been compared with 78 patients without stroke.
- Plasma was withdrawn from each patient through centrifugation of 1500 g of blood samples.
- The measurement of PARK7 and NDKA was performed by ELISA.
- Nonparametric tests were conducted to discriminate both biomarkers.

**Results**

- PARK7 and NDKA are expressed early in patients with stroke and decrease to baseline value on day 14 after admission.
- The receiver-operating characteristic curves determined a great area under the curve for PARK7 than NDKA in stroke group but also in groups of different types of stroke (ischemic stroke, hemorrhagic stroke or subarachnoid hemorrhage).
- Their expression are respectively similar in either ischemic stroke, hemorrhagic stroke and subarachnoid hemorrhage.
- At a given time point, PARK7 and NDKA levels do not correlate with National Institute for Health Stroke Scale score value.

**Discussion**

- Results of this study are complementary to latest findings: PARK7 has better diagnostic value for acute stroke than NDKA.
- PARK7 may be more able to identify stroke patients that are more likely to benefit from early therapies.
- Both markers have limitations; they are similarly expressed in ischemic stroke and hemorrhagic stroke, so they are not able to specify stroke type.
- The limitation of this study may be its small sample size but its insight announces the superiority of PARK7 over NDKA.

**Conclusion & future perspective**

- PARK7 is more accurate for diagnosis of patients in acute stroke and its usefulness will be improved in the future research.
- Biomarkers in stroke are a developing field. With further studies, biomarkers may improve the ability to diagnose stroke, predict cause and certain complications, and stratify patients to treatment.
- The development of more accurate biomarkers (such as PARK7) will help early triage of stroke patients requiring timely treatment.
